# Prevalence of *Chlamydia trachomatis* in the general population in Germany – a triangulation of data from two population-based health surveys and a laboratory sentinel system

**DOI:** 10.1186/s12889-022-13456-7

**Published:** 2022-06-03

**Authors:** Martyna Gassowski, Christina Poethko-Müller, Martin Schlaud, Andrea Sailer, Kerstin Dehmel, Viviane Bremer, Sandra Dudareva, Klaus Jansen, Chlamydia trachomatis laboratory sentinel team, Michael Baier, Eberhard Straube, Armin Baillot, Patricia Bartsch, Thomas Brüning, Josef Cremer, Helga Dallügge-Tamm, Arndt Gröning, Stephan Eicke, Dagmar Emrich, Gundula Fritsche, Rosi Gjavotchanoff, Peter Gohl, Matthias Götzrath, Axel Meye, Ingrid Ehrhard, Beate Köpke, Birgit Henrich, Caroline Kastilan, Susanne Lehmann, Anneliese Märzacker, Bernhard Miller, Gerrit Mohrmann, Christian Pache, Roland Pfüller, Carsten Tiemann, Hilmar Wisplinghoff, Thomas Müller, Christian Aepinus

**Affiliations:** 1 Postgraduate Training for Applied Epidemiology (PAE, German Field Epidemiology Training Programme), Robert Koch Institute, Berlin, Germany; 2 Department for Infectious Disease Epidemiology, Unit of Gastrointestinal Infections, Zoonoses, and Tropical Infections, Robert Koch Institute (RKI), Berlin, Germany; 3 Department of Epidemiology and Health Monitoring, Unit for Physical Health, Robert Koch Institute, Berlin, Germany; 4 Department of Epidemiology and Health Monitoring, Central Epidemiological Laboratory, Robert Koch Institute, Berlin, Germany; 5 Department for Infectious Disease Epidemiology, Unit for HIV/AIDS, STI and Blood-Borne Infections, Robert Koch Institute, Berlin, Germany

**Keywords:** Chlamydia trachomatis, Sexually transmitted infections, Screening, Prevalence

## Abstract

**Background:**

*Chlamydia trachomatis* (chlamydia) is a common, frequently asymptomatic, sexually transmitted infection. It can result in severe sequelae, such as ectopic pregnancy and infertility. In Germany, chlamydia is not notifiable. An opportunistic screening program for women < 25 years was introduced in 2008. The aim of this research was to triangulate different data sources to describe the epidemiological situation of chlamydia in Germany and to investigate whether the current target group of the chlamydia screening program aligns with these findings.

**Methods:**

Urine specimens from participants from population-based health examination surveys of children (2014–17) and adults (2008–11) were tested for chlamydia, using nucleic acid amplification testing. These data were used to generate weighted chlamydia prevalence estimates by age group and sex. Data from a nationwide chlamydia laboratory sentinel system (2014–16) were used to calculate the positive proportion among individuals tested for chlamydia by age, sex and test reason.

**Results:**

Using data from the population-based surveys, we found a chlamydia prevalence estimate of 2.8% (95% confidence interval (CI) 1.0–7.5%) among all 15- to 17-year-old girls and of 9.6% (95% CI 0.0–23) among those reporting to be sexually active. In adult women, we found the highest prevalence among 18- to 24-year-olds (all: 2.3%; 95% CI 1.0–5.3%; sexually active: 3.1%; 95% CI 1.3–7.0%). In adult men, we found the highest prevalence among 25- to 29-year-olds (all: 3.5%; 95% CI 1.6–7.7%; sexually active: 3.3%; 95% CI 1.3–7.8%). Data from the chlamydia laboratory sentinel showed the highest positive proportion among those opportunistically screened in 19-year-old women (6.1%; 95%- CI 5.9–6.4%), among those screened due to pregnancy in 15-year-old girls (10%; 95% CI 8.5–12%), and among those tested due to symptoms or a positive partner in 19-year-old women (10%; 95% CI 9.8–11%) and 19-year-old men (24%; 95% CI 22–26%).

**Conclusions:**

Chlamydia seems to mainly affect adolescents and young adults in Germany, with similar overall prevalence in men and women, but with slightly different age distributions. Women at highest risk of chlamydia are covered by the current screening program but given the on-going discussions in high-income countries on cost-effectiveness and benefit-to-harm ratio of these programs, the program-aim needs reconsideration.

## Background

*Chlamydia trachomatis* (referred to as chlamydia) is one of the world’s most common sexually transmitted infections. According to the most recent estimation of the World Health Organization, approximately 128 million new cases of chlamydia occurred globally in 2020 [1] and the highest chlamydia prevalence is observed among adolescents and young adults [2, 3]. Chlamydia infections are frequently asymptomatic and can therefore remain undiagnosed; it is assumed that one in two infected men and four in five infected women do not develop any symptoms [4]. The infection can resolve spontaneously but in cases where it persists, it can lead to severe sequalae. Women, once infected with chlamydia, have an increased risk of developing pelvic inflammatory disease (PID), which in turn can lead to ectopic pregnancy and tubal factor infertility [5]. In a modelling exercise, Price et al. estimated the probability to develop clinical PID following an untreated incident episode of chlamydia to be 16% [6]. At the same time, a diagnosed infection can easily be treated with antibiotics such as doxycycline or azithromycin [7].

Chlamydia is not notifiable in Germany and its epidemiology and development over time in the German context are therefore not clear. An exception to this is the federal state of Saxony (covering 5% of the German population), where chlamydia surveillance is undertaken locally. Here, the chlamydia notification rates have been stable over the years 2016–2019 at around 100/100,000 population, with a slight increase in 2021 [8].

Most patients with a test indication for chlamydia in Germany will traditionally attend a specialized physician such as a gynaecologist, urologist or dermatologist, but a family doctor can also order a test. In many larger cities it is also possible to get tested at the local public health authority (LPHA), but this is less common in smaller cities and rural areas, as LPHA are not obliged to offer this service.

In 2008, an opportunistic annual screening of sexually active women < 25 years of age was introduced in Germany. Gynaecologists are the main provider of the screening offer, as there are no sexual health clinics in Germany and family doctors are not eligible for reimbursement of screening tests through this program. The screening test is a test of urine, using nucleic acid amplification testing (NAAT). Since its introduction, the screening coverage increased from 8 to 12%, where it is thought to have stabilized [9]. There is also a screening program for pregnant women since 1995, which was expanded in 2008 to also cover women going through a planned abortion.

At the time the opportunistic screening program was introduced, limited data on chlamydia prevalence were available to inform the age limit. In this study we attempted to describe the prevalence and distribution of the infection across the population using alternative data sources, in order to fill this knowledge gap. We therefore formulated the aims of our analysis as follows: 1) to estimate the prevalence of chlamydia in the general population and to calculate positive test proportions among individuals tested for chlamydia using data from national, population-based health surveys and a chlamydia laboratory sentinel system for chlamydia and 2) to investigate whether our findings align with the current target group of the national chlamydia screening program.

## Methods

We used data from three data sources: a population-based health examination survey of children and adolescents, a population-based health examination survey of adults, and a nationwide laboratory sentinel system for chlamydia. The two surveys are part of a national health monitoring system and consist of both cross-sectional and longitudinal study populations. In this study, we analysed the cross-sectional sample of each survey, from which urine samples had been collected. All methods of the two surveys and of the laboratory sentinel system from chlamydia were carried out in accordance with all relevant guidelines and regulation.

### Health examination survey of children and adolescents

The most recent health examination survey of children and adolescents (KiGGS) took place 2014–2017. Using two-stage cluster sampling, participants were selected to form a representative sample of uninstitutionalized individuals aged 0–17 years with a permanent residency in Germany. Details regarding the methodology of the survey can be found elsewhere [10]. Parent-administered questionnaires were used to collect data on all participants, whereas 11- to 17-year-old participants additionally filled in a self-administered questionnaire. Both questionnaire were filled in at home. The questions contained in the self-administered questionnaires varied slightly depending on the age of the participants. For participants aged 14–17 years, the questionnaire included a question on whether the participant had already had sexual intercourse, which could be answered by either yes or no.

A random subsample was selected for physical examinations, which included the collection of a urine sample. Retained urine samples were stored at -80 °C. Frozen urine specimens of male and female participants aged 15–17 years were defrosted and tested for chlamydia by polymerase chain reaction (PCR), using the Aptima *Chlamydia trachomatis* Assay from Hologic in December 2017. As the testing was performed retrospectively, results were not returned to the participants. Samples from 14-year-olds were not tested for chlamydia as very low case numbers were expected in this age group, which with the given sample size would result in highly imprecise prevalence estimates.

### Health examination survey of adults

The most recent health examination survey of adults (DEGS1) took place in 2008–2011. Participants were selected using a complex probability-based two-stage cluster design, that has been described in detail elsewhere [11]. The target population for the cross-sectional sample of the survey were uninstitutionalized adults aged 18–79 years with a permanent residency in Germany. As part of the health examination, a urine sample was collected. Again, retained urine samples were stored at -80 °C and later defrosted and tested for the presence of chlamydia by PCR, using the Aptima *Chlamydia trachomatis* Assay from Hologic in December 2017. Due to the retrospective nature of the testing, results were not returned to the participants. All participants were asked about the number of sexual partners in the last 12 months; the values of this variable were recoded to “0 partners” or “1 or more partners” and the variable was consecutively interpreted as sexual activity in the last 12 months (yes or no).

### *Chlamydia trachomatis* laboratory sentinel system

The *chlamydia trachomatis* laboratory sentinel system collected data from laboratories on all chlamydia tests performed, including information on test results, test reasons, patients’ age and sex. The laboratory sentinel system was estimated to cover approximately 1/3 of all chlamydia tests performed in Germany, a detailed description of this system has been published elsewhere [12, 13]. All tests can be grouped by three test reasons: screening test for women < 25 years, screening test for pregnant women (this also includes women going through a planned abortion) and test due to symptoms or to a positive test of a sexual partner (applicable for both men and women). For the screening programs, first void urine is used and tested using NAAT. For patients being tested due to symptoms or a positive partner, the type of sample and test is decided by the treating physician and therefore varies. For the current analysis, data collected in years 2014–2016 from patients aged 15–39 years were used.

### Statistical analysis

Weighted prevalence estimates and 95% confidence intervals (95% CI) by sex and age group for all participants and only sexually active participants were generated from the survey data on adults and adolescents using the svy-command in Stata. Participants with missing information on sexual activity were excluded from this part of the analysis. Weights accounting for deviations of the samples from the population structure in regard to age, sex, federal state and nationality were used in both the adult and adolescent samples. In addition, weights used for the adult sample also accounted for type of municipality and educational status, while those for the adolescent population also accounted for parental educational levels [10, 14].

Data from the chlamydia laboratory sentinel system were used to calculate the number of tests performed in the considered three-year period for each test reason by age. Additionally, positive proportions with 95% CI were calculated for each test reason by age.

All statistical analyses were performed using Stata 15 (Stata Corporation, College Station, TX; USA).

## Results

### Health examination survey of children and adolescents

In total, 700 adolescents aged 15–17 years were recruited for the examination arm of the health survey, which corresponded to a response rate of 41%. Urine samples were collected from 670 (96%) participants and 619 (88%) chlamydia test results were available. Of these, 8 samples were positive, 7 from girls and 1 from a boy (Table 1). Applying survey weights, this resulted in prevalence estimates of 2.8% (95% CI 1.0%-7.5%) for girls and 0.1% (95% CI 0.0%-0.7%) for boys. Questionnaire data were available for 605 (98%) tested participants, data on experience of sexual intercourse was available for 572 (92%). All 47 participants with a missing value on sexual intercourse were tested negative for chlamydia. Prevalence estimates in the subpopulations of sexually experienced adolescents were 9.6% (95% CI 0.0%-23%) for girls and 0.5% (95% CI 0.1%-3.4%) for boys.

**Table 1:** Weighted chlamydia prevalence estimates in adolescent girls and boys (15–17 years), based on data from health examination survey of children in Germany (KIGGS, 2014–17)

	**All study participants**			**Ever sexually active participants**^**a**^		
	*Positive participants/ all participants* *(n/N)*	*Weighted point estimate (%)*	*95% confidence interval (%)*	*Positive participants/ all participants (n/N)*	*Weighted point estimate (%)*	*95% confidence interval (%)*
***Girls, 15–17 years***	*7/336*	*2.8*	*1.0–7.5*	*7/106*	*9.6*	*0.0–23*
***Boys, 15–17 years***	*1/275*	*0.1*	*0.0–0.7*	*1/60*	*0.5*	*0.1–3.4*

^a^ Information on sexual activity missing for 47 participants with a negative result

### Health examination survey of adults

A total of 7115 adults were recruited for the survey. This corresponded to a response proportion of 42% among first-time recrutees and 64% among follow-up participants. Urine samples were collected from 7073 (99%) participants and 6799 (96%) samples were tested for chlamydia. In total, 47 samples were tested positive, 21 from women and 26 from men (Table 2). The highest prevalence estimate among women was found in the group of 18- to 24-year-olds (2.3%; 95% CI 1.0%-5.3%), followed by 30- to 34-year-olds and 25- to 29-year-olds. In all older age groups, prevalence estimates were ≤ 1.0%. Information on sexual activity was missing for 98 women, all of whom had a negative test result. When considering only those women who reported sexual activity in the last 12 months, the estimate for 18- to 24-year-olds increased to 3.1% (95% CI 1.3–7.0%). Among men, the highest prevalence estimate was found among 25- to 29-year-olds (3.5%; 95% CI 1.6%-7.7%), followed by 30- to 34-year-olds and 18- to 24-year-olds. The prevalence estimates in older age groups decreased with increasing age. Information on sexual activity in the last 12 months was missing for 142 men, two of whom had a positive test result. Among men who reported sexual activity in the last 12 months, the prevalence estimate of the 18- to 24-year-olds increased to 2.2% (95% CI 0.9–5.2%) while that of 25- to 29-year-olds decreased to 3.3% (95% CI 1.3–7.8%).

**Table 2:** Weighted chlamydia prevalence estimates in adult women and men (18–79 years), based on data from health examination survey of adults in Germany (DEGS1, 2008–11)

		**All participants**			**Participants sexually active** **in the last 12 months**^**b**^		
*Sex*	*Age groups (years)*	*Positive participants/ all participants (n/N)*	*Weighted point estimate (%)*	*95% confidence interval (%)*	*Positive participants/ all participants (n/N)*	*Weighted point estimate (%)*	*95% confidence interval (%)*
**Women**	*18–24*	7/290	2.3	1.0–5.3	7/229	3.1	1.3–7.0
	*25–29*	4/224	1.2	0.4–3.5	3/192	0.9	0.3–3.2
	*30–34*	2/181	1.5	0.3–7.2	2/158	1.7	0.3–8.4
	*35–39*	0/228			0/194		
	*40–44*	1/296	0.8	0.1–5.3	1/259	0.9	0.1–5.9
	*45–49*	2/357	0.6	0.1–3.0	2/306	0.7	0.1–3.7
	*50–54*	2/409	0.3	0.1–1.3	2/312	0.4	0.1–1.8
	*55–59*	1/325	0.3	0.0–1.9	1/226	0.4	0.1–2.8
	*60–64*	1/337	1.0	0.1–6.8	1/186	1.9	0.3–12.1
	≥ *65*	1/888	0.0	0.0–0.4	1/324	0.1	0.0–1.0
	***Total***	**21/3,535**	**0.7**	**0.4–1.2**	**20/2,386**	**1.0**	**0.6–1.6**
**Men**	*18–24*	6/299	1.9	0.8–4.2	5/230	2.2	0.9–5.2
	*25–29*	9/197	3.5	1.6–7.7	8/169	3.3	1.3–7.8
	*30–34*	2/183	2.1	0.5–8.4	2/159	2.5	0.6–9.7
	*35–39*	2/205	1.0	0.2–4.0	2/185	1.1	0.3–4.6
	*40–44*	2/261	1.1	0.3–4.5	1/225	0.8	0.1–5.6
	*45–49*	0/316			0/269		
	*50–54*	2/323	0.7	0.2–2.7	2/279	0.8	0.2–3.2
	*55–59*	1/294	0.2	0.0–1.1	1/241	0.2	0.0–1.3
	*60–64*	0/299			0/235		
	≥ *65*	2/887	0.2	0.0–1.3	0/516		
	***Total***	**26/3,264**	**1.0**	**0.6–1.5**	**21/2,508**	**1.0**	**0.6–1.7**

^b^ Information on sexual activity missing for 98 women (all negative for chlamydia) and 142 men (2 positive for chlamydia)

### Chlamydia laboratory sentinel system

**Fig. 1:**
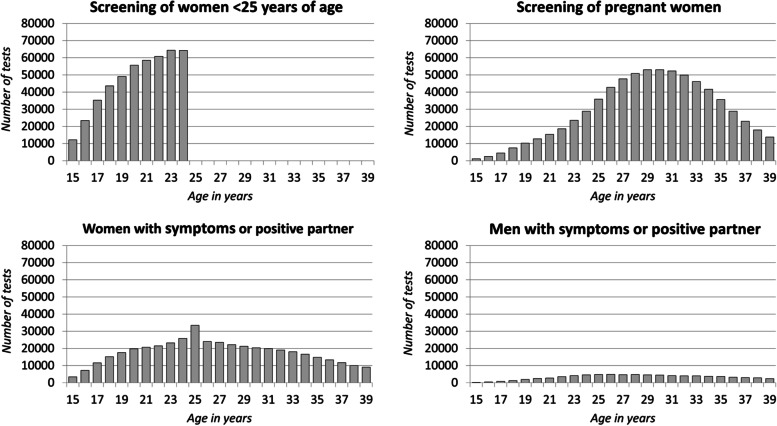
Number of chlamydia tests by test reason, as recorded through the chlamydia laboratory sentinel system in Germany, 2014–16

**Fig. 2:**
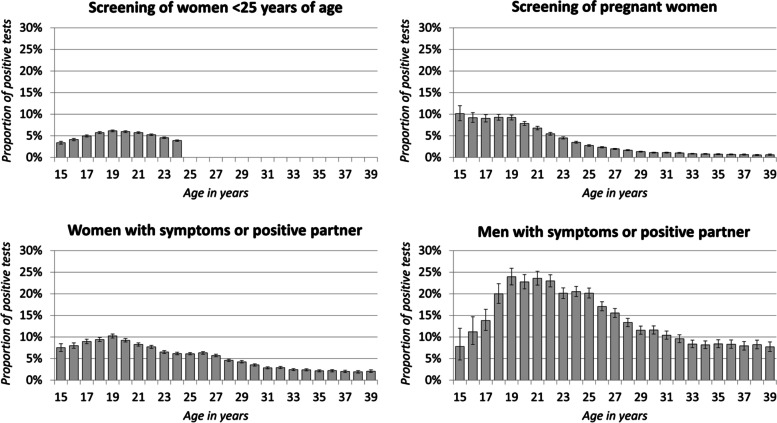
Proportion (point estimates and 95% CI) of positive chlamydia tests by test reason, as recorded through the chlamydia laboratory sentinel system in Germany, 2014–16

In the period 2014–2016, the chlamydia laboratory sentinel network reported 1 717 081 tests with a known test reason. The majority of tests (42%) were screening tests of pregnant women (including women planning an abortion), 27% were screening tests of women < 25 years of age and 31% were tests due to symptoms or a positive sexual partner, with over 4/5 of all tests being performed in women. The number of screening tests for women < 25 years of age steadily increased by age (Fig. 1). The highest proportion positive for chlamydia in this group was found among 19- and 20-year-olds (6.1%; 95% CI 5.9–6.4% and 6.0%; 95% CI 5.8–6.2%, respectively), while the lowest positive proportions were found in the youngest (15-year-olds) and oldest (24-year-olds) age groups (3.4%; 95% CI 3.1–3.7% and 3.9%; 95% CI 3.7–4.0%, respectively) (Fig. 2).

The highest numbers of screening tests among pregnant women were performed in 29-, 30- and 31-year-olds, with more than 52 000 tests performed. The highest positive proportion was found in 15-year-olds at 10% (95% CI 8.5–12%). In age groups 16- to 19-years, positive proportions above 9% were found.

The number of tests performed in women due to symptoms or a chlamydia-infected partner increased with age up to the age of 25 years and decreased thereafter. The positive proportion in 15-year-olds was 7.5% (95% CI 6.7–8.5%) and peaked at 10% (95% CI 9.8–11%) in 19-year-olds. Thereafter, the positive proportion decreased with age and stabilized at approximately 2%.

The number of tests performed in men due to symptoms or a positive sex partner ranged from 231 in 15-year-old boys, up to 4 946 in 26-year-old men, whereafter it slowly decreases to 2 397 tests of 39-year-olds. A sharp increase in the positive proportion was observed in 15- to 19-year-olds, going from 7.8% (95% CI 4.7–12%) to 24% (95% CI 22–26%). Thereafter, the positive proportion decreased with age, stabilizing at approximately 8%.

## Discussion

By triangulating three different data sources, we attempted to describe the distribution of chlamydia in the general population in Germany and to investigate whether our findings align with the current target group of the opportunistic screening program. Data from all three sources suggest that chlamydial infections are most prevalent in adolescents and young adults, with the prevalence peaking slightly later in men as compared to women. Overall, we found no difference in the prevalence between men and women in the adult population but observed a higher prevalence in adolescent girls than in boys, albeit this finding is based on low numbers. The main strength of this study is that it provides chlamydia prevalence estimates and thus fills an important gap, given that chlamydia is not a notifiable infection in Germany.

The absence of an apparent difference in prevalence between the sexes supports the conclusion that the prevalence of chlamydia infection in men and women appears to be more similar than dissimilar, as drawn by Dielissen et al. from a review of literature [15]. An exception to this were adolescents (15-to 17-year-olds), where girls seemed more affected than boys. Age-bridging could be an explanation for this discrepancy, as adolescent girls are more likely to engage in sexual activity with an older, and thus potentially already exposed (infected) partner, than boys are [16].

Young age is a known risk factor for chlamydia and so our findings were expected and in line with those from other countries [2, 17, 18]. A closer look at the prevalence in the younger age groups deriving from the two data sources suggests a slightly different age distribution. While the chlamydia prevalence estimated using the health examination survey data suggests the highest prevalence in the youngest age group, the proportion positive found through the laboratory sentinel system was increasing with age among the teenagers and peaked in the group of 19-year-olds. This discrepancy is likely due to the different nature of the data sources. In contrast to the prevalence estimates generated from the population-based health examination surveys, the positive proportions calculated from the chlamydia laboratory sentinel data are not representative for the general population in Germany and need to be interpreted in the context in which the samples were taken (i.e. the reason for testing). In order to receive a screening test for chlamydia, it is required that 1) a woman attends a gynaecologist and 2) the gynaecologist offers a test. The proportions of sexually active women not attending a gynaecologist and of consultations of eligible women, where no test was offered, are unknown, but are likely to vary by age. One estimate suggests that 54% of 14- to 17-year-old girls in Germany have ever attended a gynaecological practice, but the proportion of sexually active girls who have not is not known [19]. It is possible that the screening program misses some parts of this important group and that the data from the chlamydia laboratory sentinel surveillance system thus underestimate the proportion infected.

Our data from the pregnancy screening also suggest that chlamydia is more common among the younger age groups, with positive proportions of 9%-10% among adolescent girls in this population. As opposed to older women, where stable and monogamous partnerships with a low risk of chlamydial infection are increasingly likely, a pregnancy at young age is more likely to be unintended and rather another marker of risky behaviour. Although this subgroup may have more risky behaviour than sexually active teenagers overall, the similar chlamydia prevalence estimate that we found in sexually active adolescent girls (9.6%, 95% CI 0.0–23%) in the health examination survey, though with a wide confidence interval, could be suggesting that the chlamydia prevalence among sexually active girls in this age group is high.

Naturally, the positive proportions among those who were tested due to symptoms or a positive partner were high, as these individuals had an indication to be tested, as opposed to those who were screened. It can be assumed that only a small proportion in this group was tested due to a positive partner, as the recommendation in Germany in this case is to treat without prior testing [20]. Although the positive proportions cannot directly be compared to the prevalence estimates from the health examination surveys, the distributions across age groups are similar in both men and women, with the highest positive proportions in 19- to 22-year-old men and 17- to 20-year-old women. The higher proportions found in the younger age groups of those tested due to symptoms suggest that chlamydia circulates more in these groups, whereas urogenital symptoms in older age groups seem to rather have other causes. This is also in accordance with findings from a study of chlamydia prevalence in individuals seeking HIV testing at local public health authorities in North-Rhine Westphalia (the largest federal state in Germany), where the prevalence in women and heterosexual men was highest in the 18- to 24-year-olds [21].

The screening program does in theory target the group of women with the highest risk of chlamydia but its low coverage means that the vast majority of eligible women are not reached [12]. However, as a substantial reimbursement for doctors for the pre-test counselling of women participating in the screening program has been introduced after the period studied (April 2020), this might have led to an increase in coverage. The screening uptake may also be influenced by the poor level of knowledge about chlamydia in the general population, as suggested by the German National Sex Survey, a representative survey of sexual behaviours, attitudes and lifestyles of the general population from 2018–19 [22]. An educational campaign on chlamydia and chlamydia screening targeting both the general public and medical practitioners was launched in October 2021 by the German Federal Center for Health Education. Hopefully, this campaign will lead to improved screening coverage.

Opportunistic screening appears to be most appropriate in the younger age groups, as we did observe a decreasing prevalence with age. However, risk-based screening may be an option to explore for older age groups, as they are also affected. Further research of this population would be advised before formulating screening criteria.

There are ongoing discussions about the effectiveness of chlamydia screening programs regarding different endpoints such as lowering chlamydia prevalence, preventing PID, ectopic pregnancy or female infertility, or the cost effectiveness of implementing appropriate measures to reach these goals [23–27]. Expert panels have recently pointed out that it is not possible to assess the effectiveness of such measures in a universal way, as the same measures may lead to different results in various settings, depending much on the local context and the specific national conditions and goals [28, 29]. Our analysis did not aim to evaluate the effectiveness of the German chlamydia screening program to prevent the named sequelae as such, as there are not sufficient data available, but rather to investigate whether this program appropriately targets the group with the highest chlamydia prevalence in Germany.

There are several important limitations to this analysis and to the various data sources used that should be considered. The numbers of study participants who tested positive were low in both health examination surveys, partly resulting in large confidence intervals. The chlamydia laboratory sentinel system collects data on the number of tests performed, rather than number of persons tested. Another analysis of the sentinel data found that 23.1% of women and 11.9% of men had been tested more than once within a seven-year-period [30]. The different years in which data from the various data sources were collected are a further limitation, making them not directly comparable. However, unpublished data from the chlamydia laboratory sentinel system suggest that there has been little change in the proportions of women testing positive for chlamydia over time (personal communication). The use of urine samples rather than vulvo-vaginal swabs in women may have led to an underestimation of chlamydia in both the survey samples and the screened populations. It has been established that for women, a swab is the preferred specimen due to higher sensitivity in comparison to urine samples [7]. Test sensitivity may also have been reduced through the practice of pooling of up to five samples, which was relatively common in the first years of the screening program. Furthermore, urine samples were collected in the surveys without any instructions (e.g. midstream or first void) and most samples were stored for several years before being tested, both of which may have impacted negatively on sample quality. It can therefore not be excluded that some infections may have been missed and that our estimates are an underestimation of the true chlamydia prevalence.

## Conclusions

Chlamydia seems to mainly affect adolescents and young adults in Germany, with similar overall prevalence in men and women, but with slightly different age distributions. As chlamydia is not a notifiable infection in Germany, these findings offer important insight into the epidemiological situation in the country and will be relevant in guiding future prevention strategies.

The target group of the current screening program does include the age groups of women with the highest risk of chlamydia, but a cost-effectiveness study would be needed to determine exact lower and upper age limits, as we did not observe any clear cut-off points. At the same time, the screening program disregards the male half of the population, where we found an equally high overall prevalence.

The German public health decision makers on STI prevention should closely follow and engage in the current discussions of the benefit-to-harm ratio and the cost-effectiveness of widespread testing for asymptomatic chlamydia infections in high-income countries, as the resulting conclusions might lead to a fundamental change in approach, which may well impact the future of the screening program.

## Data Availability

The authors confirm that some access restrictions apply to KiGGS and DEGS1 data underlying the findings. The data sets cannot be made publicly available because informed consent from study participants did not cover public deposition of data. However, the data underlying the findings is archived in the 'Health Monitoring' Research Data Centre at the Robert Koch Institute (RKI) and can be accessed by researchers on reasonable request. On-site access to the data set is possible at the Secure Data Center of the RKI’s 'Health Monitoring' Research Data Centre. Requests should be submitted to the 'Health Monitoring' Research Data Centre, Robert Koch Institute, Berlin, Germany (e-mail: fdz@rki.de). *Chlamydia trachomatis* laboratory sentinel: The datasets used and/or analysed during the current study are available from the corresponding author on reasonable request.

## Abbreviations

PID: Pelvic inflammatory disease
NAAT: Nucleic acid amplification test
KiGGS: Health examination survey of children and adolescents in Germany (German: Studie zur Gesundheit von Kindern und Jugendlichen in Deutschland)
PCR: Polymerase chain reaction
DEGS1: Health examination survey of adults in Germany (German: Studie zur Gesundheit Erwachsener in Deutschland)
95% CI: 95% Confidence interval
